# Modulation of Master Transcription Factor Expression of Nile Tilapia Leukocytes via Cholinergic Pathways

**DOI:** 10.3390/ijms262211206

**Published:** 2025-11-20

**Authors:** Manuel Ivan Girón-Pérez, Kenia María Ramírez-Ibarra, Carlos Eduardo Covantes-Rosales, Daniel Alberto Girón-Pérez, Francisco Fabián Razura-Carmona, Arturo Contis-Montes de Oca, Jorge Morales-Montor, Lenin Pavón, Gladys Alejandra Toledo-Ibarra

**Affiliations:** 1 Laboratorio Nacional de Investigación para la Inocuidad Alimentaria (LANIIA)—Unidad Nayarit, Universidad Autónoma de Nayarit, Calle Tres S/N, Colonia, Cd. Industrial, Tepic Z.C. 63000, Mexico; ivangiron@uan.edu.mx (M.I.G.-P.); carlos.covantes@uan.edu.mx (C.E.C.-R.);; 2 Plan de San Luis y Díaz Mirón s/n, Sección de Estudios de Posgrado, Escuela Superior de Medicina, Instituto Politécnico Nacional, Col. Casco de Santo Tomas, Alcaldía Miguel Hidalgo, Ciudad de México Z.C. 11340, Mexico; neocontis@hotmail.com; 3 Departamento de Inmunología, Instituto de Investigaciones Biomédicas, Universidad Nacional Autónoma de México, Coyoacán, Ciudad de México Z.C. 04510, Mexico; jmontor66@iibiomedicas.unam.mx; 4 Laboratorio de Psicoinmunología, Instituto Nacional de Psiquiatría Ramón de la Fuente Muñiz, Ciudad de México Z.C. 14370, Mexico; lkuriaki@inprf.gob.mx

**Keywords:** non-neuronal cholinergic system, acetylcholine, acetylcholine receptor, pesticide

## Abstract

Teleost fish are the first evolutionary group to exhibit an innate and adaptive immune system. Within the mechanisms of adaptive immunity, fish possess, among others, T-helper cells (CD4-like) and their differentiation machinery, regulated by the master transcription factors T-bet, GATA3, Foxp3, and RORγ. Many studies support the existence of a non-neuronal cholinergic system involved in the immune response, named after the ability of leukocytes to synthesize de novo acetylcholine (ACh). Organophosphorus pesticides (OPs), such as diazoxon (DXN), are examples of compounds that act as cholinergic disruptors with immunotoxic effects. The present study aimed to evaluate the expression of transcription factors in leukocytes (spleen mononuclear cells, SMNCs) of Nile tilapia by modulating cholinergic pathways in immune cells using agonists, antagonists, and diazoxon (DXN), an anticholinesterase substance. The obtained data showed a significant increase in RORγ mRNA expression upon stimulation with the nicotinic agonist, whereas activation of the muscarinic receptor with its agonist increased T-bet mRNA expression. An alteration in RORγ expression levels induced by DXN exposure was also observed. The results suggest a probable directing of the immune response towards a pro-inflammatory profile orchestrated mainly by RORγ and T-bet transcription factors in response to cholinergic stimuli.

## 1. Introduction

Teleost fish are among the earliest diverging groups of vertebrates, with fully developed innate and adaptive immune responses [1]. In addition to a robust innate immune system, teleosts have essential components of adaptive immunity, including cellular and humoral elements such as B lymphocytes, T lymphocytes and their subpopulations, as well as immunoglobulin isotypes (IgM, IgD, and IgZ) [2,3,4,5].

Likewise, in these organisms, the cell-mediated immune response is governed by different types of leukocytes, including cytotoxic T lymphocytes (CD8-like) and helper T cells (CD4-like) [6,7]. CD4-expressing helper T cells (CD4^+^ Th cells) coordinate the immune response by acting as effector cells or memory cells. In teleosts, the function of these cells is believed to be similar to that in mammals, given their molecular similarities [1]. After CD4^+^ cell activation, naïve cells can differentiate into subsets such as Th1, Th2, Th17, and regulatory T cells (Tregs), with each subpopulation associated with its cytokine production profile [8].

Th1 cell differentiation involves several transcription factors, including the master transcription factor T-bet and the signal transducers and activators of transcription-1 (STAT-1) and STAT-4 [9,10,11,12]. Additionally, GATA3 has been identified and isolated in various fish species and is known to play a crucial role in differentiation toward the Th2 cell lineage [13,14].

Th17 cells are characterized by producing IL-17A and IL-17F (pro-inflammatory cytokines), and their expression is regulated by RORγ, RORα, and STAT3 [15]. Orthologs of RORγ and isoforms of IL-17A/F have been identified in teleost fishes [16,17,18]. Finally, the differentiation of IL-10-secreting regulatory T cells, responsible for maintaining self-tolerance and immune homeostasis (Treg) [19], occurs through the transcription factor Forkhead box P3 (FoxP3) [20,21,22].

The involvement of the cholinergic pathway in immune response is explained by events in the known non-neuronal cholinergic system. Numerous studies support the existence of a non-neuronal cholinergic system in immune cells [23,24]. Since leukocytes possess all the biochemical and molecular components needed to synthesize de novo acetylcholine (ACh), these components include choline acetyltransferase (ChAT), muscarinic and nicotinic ACh receptors (mAChRs and nAChRs, respectively), acetylcholinesterase (AChE), and other molecules involved in acetylcholine metabolism. Moreover, ACh may influence immune function through various mechanisms, such as T-cell activation, antigen-specific antibody production, and cytokine production, among others [23,24,25,26].

Organophosphate pesticides (OPs) are examples of compounds that function as cholinergic disruptors. Diazoxon (DXN), a metabolite of the OP diazinon, is phosphoric acid diethyl 6-methyl-2-(1-methylethyl)-4-pyrimidinyl ester. Its primary mechanism of toxicity is the AChE inhibition, which leads to ACh accumulation and, subsequently, overstimulation of mAChRs and nAChRs [27,28]. Immunotoxic effects are now recognized in non-target organisms such as vertebrates, including fish, where studies report cellular and humoral changes resulting from exposure to this type of compound [29,30,31,32].

The aforementioned suggests the possibility of modulating the immune response by manipulating cholinergic receptors (mAChR or nAChR) on immune cells with agonists, antagonists, and an anticholinesterase agent. Therefore, this study aimed to evaluate the expression of immune master transcription factors (T-bet, GATA3, Foxp3, and RORγ) in Nile tilapia leukocytes exposed to cholinergic agonists (nicotine, carbachol), antagonists (bungarotoxin and atropine), and an AChE inhibitor (DXN).

## 2. Results and Discussion

### 2.1. Characterization of SMNCs by Flow Cytometry

**Figure 1:**
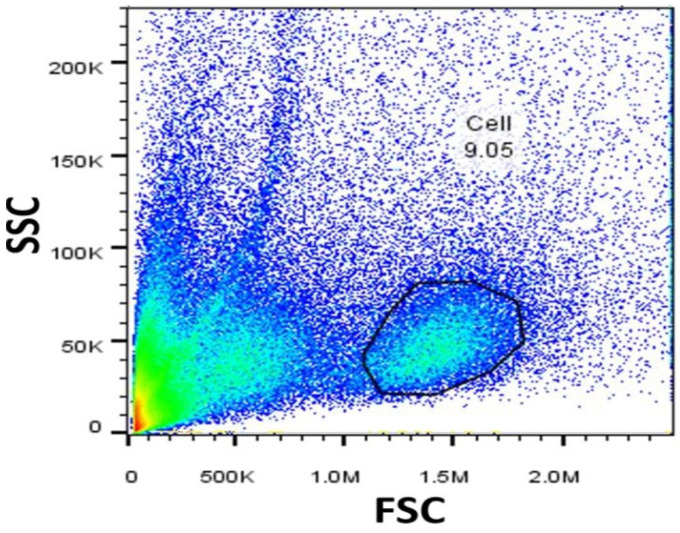
Nile tilapia spleen mononuclear cells. SMNCs characterized by flow cytometry through size (FSC) and cellular complexity (SSC).

Before evaluating the parameters, the SMNCs populations were identified using forward scatter (FSC) and side scatter (SSC) in the flow cytometer (Figure 1).

### 2.2. Expression of Transcription Factors in SMNCs Exposed to Cholinergic Agonists and Antagonists

#### 2.2.1. Nicotinic Pathway

**Figure 2:**
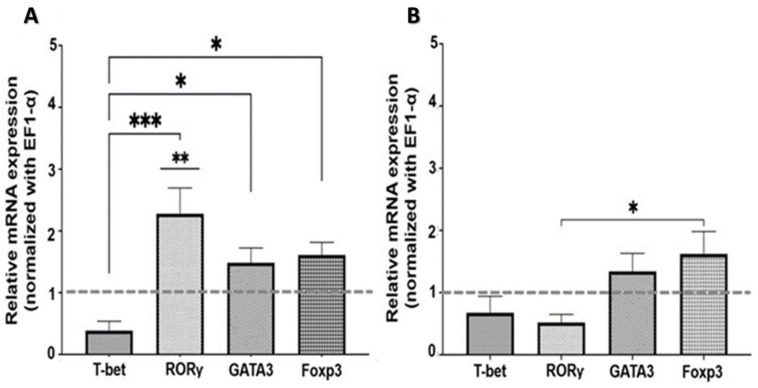
Effect of nicotinic stimulation on master transcription factor expression in SMNCs. T-bet, RORγ, GATA3, and Foxp3 mRNA levels in SMNCs exposed in vitro to nicotine (**A**) and bungarotoxin (**B**), measured by RT-qPCR (expression normalized to EF1-α). Results are shown as means ± SEM (n = 7). Differences between groups were tested with one-way ANOVA and Tukey’s multiple comparisons test. * *p* < 0.05, ** *p* < 0.01, *** *p* < 0.001.

To describe the effects of nAChR on the expression of master transcription factors, SMNCs were exposed to an nAChR agonist and an antagonist. As shown in Figure 2A, treatment with nicotine, an nAChR agonist, caused a significant increase in mRNA expression of RORγ, the master transcription factor of Th17 cells (*p* < 0.01). In contrast, there was no change in RORγ expression in the presence of bungarotoxin (nAChR antagonist) (Figure 2B). This suggests that the changes observed in RORγ expression were mediated through nicotinic receptor signaling.

The expression of T-bet mRNA, on the other hand, did not show significant differences compared to the control samples; however, it exhibits a down-regulation trend in its expression, in contrast to the other transcription factors: RORγ, GATA3, and Foxp3 (*p* < 0.001; *p* < 0.05, and *p* < 0.05, respectively) (Figure 2A).

The overexpression of RORγ could indicate a likely tendency to form the Th17-like cell lineage, driven by cholinergic signals originating from the stimulation of the nAChR.

#### 2.2.2. Muscarinic Pathway

**Figure 3:**
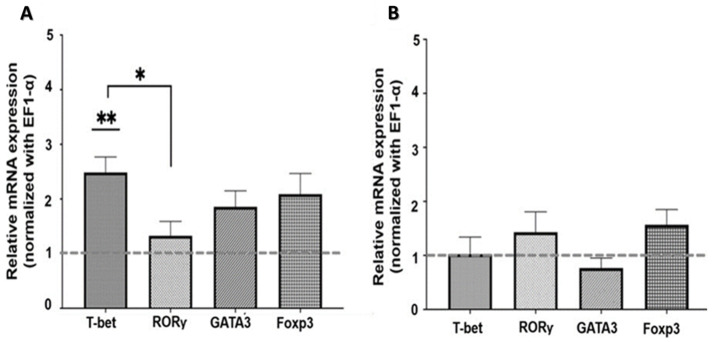
Effect of muscarinic stimulation on master transcription factor expression in SMNCs. T-bet, RORγ, GATA3, and Foxp3 mRNA levels in SMNCs exposed in vitro to carbachol (**A**) and atropine (**B**) were measured by RT-qPCR (expression was normalized to EF1-α). Results are shown as means ± SEM (n = 7). Differences between groups were analyzed using one-way ANOVA and Tukey’s multiple comparisons test. * *p* < 0.05, ** *p* < 0.01.

To assess the effects of mAChR on the expression of master transcription factors, cells were treated with both an mAChR agonist and an antagonist. The results showed a significant increase in T-bet mRNA expression (*p* < 0.001) in SMNCs exposed to the muscarinic agonist carbachol compared to the control (Figure 3A).

Exposure to atropine as an mAChR antagonist showed no significant effect on the expression of any lymphocyte master transcription factors (Figure 3B).

### 2.3. Expression of Transcription Factors in SMNCs Exposed to Diazoxon

**Figure 4:**
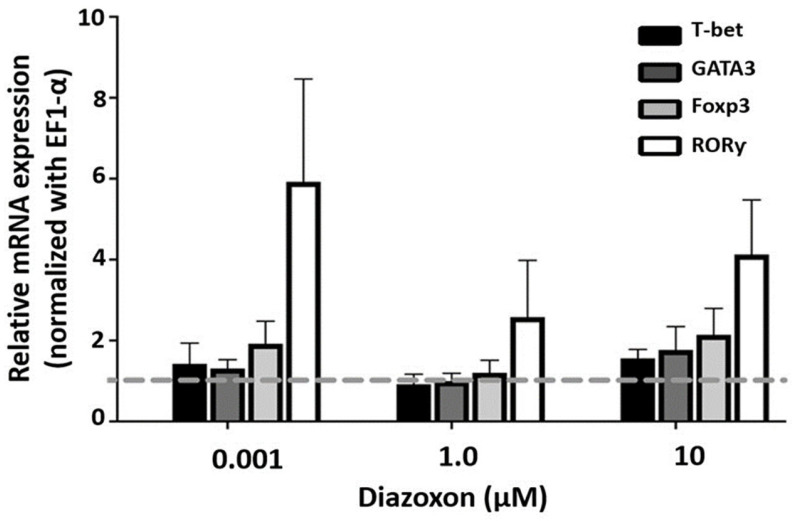
Effect of DXN on transcription factor expression in SMNCs. T-bet, RORγ, GATA3, and Foxp3 relative mRNA expression in SMNCs exposed in vitro to 0.001 μM, 1 μM and 10 μM DXN for 24 h by RT-qPCR (expression was normalized to EF1-α). Results are expressed as means ± SEM (n = 7).

An anticholinesterase compound like DXN was used to analyze the effect of cholinergic pathway disruption on the immune response. The results show no significant difference between treated groups and controls (Figure 4).

The immune system can regulate its functions through autocrine or paracrine mechanisms via cholinergic signaling pathways. The biological effects of ACh are influenced by various factors, including the specific combination of AChR subtypes expressed in cells, developmental stage, and cell differentiation [33]. Simultaneous stimulation of mAChRs and nAChRs may help maintain the balance of cellular metabolic and ionic processes, whereas asynchrony between them may allow for fine-tuning in response to signals from the central nervous system, endocrine glands, and environmental stimuli [34].

The nAChRs are ionotropic receptors that form ion channels (Na^+^, K^+^, and Ca^2+^). These receptors are pentameric, composed of α (1–10), β (1–4), γ, δ, or ε subunits, enabling a range of functions depending on their ligand affinity [35].

Once nAChRs are stimulated by ACh (or an agonist), Ca^2+^ can enter the cell either directly through ion channels or indirectly via voltage-dependent channels [29]. These receptors are located in the plasma membranes of muscle cells, neurons, and non-neuronal cells, and they are involved in neuronal growth and synapse formation during learning and memory processes. Conversely, their expression in non-neuronal cells is linked to modulation of the immune response, cell differentiation, among other functions [23,35,36,37].

Most of the components involved in mammalian Th17 cell differentiation have also been identified in several teleost fishes [22]. For Th17 lineage differentiation to occur, the transcription factor RORγ, STAT3, and specific cytokines such as transforming growth factor-β (TGF-β), IL-6, or IL-2 are essential [15].

The existence of an orthologous RORγ gene, three isoforms of IL-17A/F genes, a fish-specific cytokine called IL-17N [38,39], and signaling mechanisms involving the Jnk and STAT3 pathways in Th17 cell differentiation have been reported [40]. Retinoid-related orphan receptor gamma t (RORγt) is a member of the nuclear receptor family expressed on T cells [41], which regulates IL-17 production and directs the differentiation of pro-inflammatory Th17 cells that are critical in human autoimmune and inflammatory conditions, host defense against extracellular bacteria and fungi [42,43].

Regarding the results of the present study, the increase in RORγ mRNA expression under nicotine stimulus (but not with the stimulus of bungarotoxin) can be explained by signaling pathways described so far for mammals, where activation of nicotinic receptors triggers the Jak2/PI3K/AKT/mTOR/RORγ pathway. This pathway binds to the promoter regions of IL-17, IL-21, and IL-22 genes and induces the transcription of these cytokines [44].

There is evidence showing the binding between α1nAChR and STAT3 and demonstrating that Akt is a target of STAT3. The same study also reports that nicotine may accelerate atherogenic inflammation via α1nAChR/STAT3/Akt/mTOR signaling, as shown in ApoE^-/-^ mice as an atherosclerotic model [45].

Romstamzadeh D. et al. schematized the mechanisms through which mTOR can regulate Th17 differentiation; among them, they highlight the action of the PI3K/Akt/mTOR axis as a promoter of Th17 differentiation in a S6K1/2 kinase-dependent manner that binds RORγt and transports RORγt to the nucleus [46,47]. Another mechanism of mTOR regulation involves STAT3 in various processes; however, the direct effect on STAT phosphorylation remains unclear [48,49,50].

Among the components mentioned above, the presence of nAChR in the SMNCs and S6K1 activity in the primary adaptive immune response of Nile tilapia fish has been demonstrated [51,52], as well as the activity of the Raptor/mTORC1 axis in rockfish (*Sebastes schlegelii*) [53]. However, the specific molecular mechanisms involved in immune response polarization in fish caused by cholinergic stimulation remain unknown.

An anti-inflammatory pathway commonly described in mammals and also observed in fish involves the modulation of α7nAChR [54,55]. However, it has been suggested that nAChR activation may elicit a pro-inflammatory profile, highlighting their opposing roles in the immune response and underscoring the importance of investigating the specific signaling pathways associated with distinct receptor subunits [56].

It is remarkable that, within the obtained results, the response in RORγ mRNA expression was almost eliminated in the presence of the nAChR antagonist bungarotoxin. This could indicate that the observed changes in RORγ expression were mediated through the nAChR, as seen in studies where nicotinic antagonism modulates Th17 inhibition and decreases mTOR and STAT3 signaling [57].

In other respects, as part of the non-neuronal cholinergic system, the mAChRs are G protein-coupled receptors activated by ACh. Members of this family play key roles in various physiological functions, including regulation of heart rate, smooth muscle contraction, glandular secretion, memory formation, and regulation of the immune and inflammatory responses [58,59]. In mammals, the mAChR family consists of five highly conserved subtypes (M1-M5), with different signaling pathways being activated depending on the mAChR subtype [60]. In fish, the mAChR subtypes M2-M5A, expressed in the SMNCs of Nile tilapia, have been detected [24,61]. However, in both mammals and teleost fishes, the expression of these receptors is variable.

The use of carbachol as a muscarinic agonist in this study caused a significant increase in T-bet transcription factor mRNA levels in Nile tilapia SMNCs. The transcription factor T-bet (T-box expressed on T cells), found in mammals and teleosts [10], plays a key role in the differentiation of naive Th cells into Th1 cells, as well as in the production of IFN-γ by CD4^+^ T cells, CD8^+^ T cells, and NK cells [62,63].

It has been previously documented that M1/M5 mAChRs can regulate the production of pro-inflammatory cytokines such as TNF-α, IFN-γ (the hallmark of Th1 cells), and IL-6, at least during the early stages of an immune response, and that most inflammatory cells express functional muscarinic receptors [64]. Darby M. et al., demonstrate the critical role of cholinergic signaling via the M3 subunit of mAChR in mice for an optimal immune response to pathogens through CD4^+^ T cell activation, Th1 polarization, and IFN-γ production [65].

There is evidence supporting the use of mAChR agonists as stimulants of immune and inflammatory responses, and mAChR antagonists as inhibitors of these responses, which are frequently used as treatments for various human diseases [58]. In our study, we did not observe a significant response to treatment with the mAChR antagonist. However, we did notice a trend toward a basal expression of the T-bet gene. In this context, the signaling pathways that may lead to increased expression of this transcription factor through cholinergic manipulation remain unclear and require further investigation into the intermediates within the signaling cascade.

Our research group has confirmed that OPs act as cholinergic disruptors through their immunotoxic effects on various parameters, using teleosts as the experimental model. These effects include changes in cell proliferation, cell viability, apoptosis, phagocytosis, Ca^2+^ flux, and mitochondrial membrane potential, among others [30,66,67].

The results obtained here from OPs exposure show similar behavior to the agonistic action of nAChR on RORγ gene mRNA. Considering the influence of differential receptor expression, Toledo-Ibarra et al., observed decreased expression levels of M3, M4, and M5 mAChR and β2 nAChR in SMNCs of Nile tilapia after exposure to the same concentrations of the pesticide DXN used in this methodology [32]. Regarding intracellular signaling, data indicate that DXN leads to a decrease in cAMP concentration and an increase in basal IP3 levels, which demonstrates an effect on G protein-coupled receptors (mAChR M2 and M4). On the other hand, DXN does not affect basal levels of JAK1 and STAT3 phosphorylation, cell signaling regulated primarily by nAChR-type receptors [68]. Nevertheless, little is known about the regulation of specific genes involved in these events, particularly by transcription factors. After exposure to three concentrations of the pesticide oxon in Nile tilapia SMNCs, the results mainly showed alterations in the transcription factor RORγ, although not significantly.

Lein and Fryer, 2005, propose a direct antagonistic effect of OPs on mAChRs and an indirect effect resulting from increased ACh levels due to AChE inhibition, which leads to a reduction in receptor numbers [69]. The above could explain the predominance of DXN’s effect on RORγ expression, likely through nicotinic receptor activity rather than muscarinic stimulation.

## 3. Material and Methods

### 3.1. Experimental Fish

Male Nile tilapia fish (*O. niloticus*), weighing 250 ± 50 g, were obtained from a local farm with informed consent from the owners for research purposes only. The fish were acclimated in 400 L tanks for four weeks and fed daily with a commercial dry feed containing 25% protein (Nogafish, Ocotlán, Jalisco, Mexico). Water temperature was maintained at 26 °C ± 2 °C with constant aeration under optimal conditions (pH 8.0, dissolved oxygen 6.0 mg/L) throughout the experiment. All animal experiments were conducted in accordance with Directive 2010/63/EU of the European Parliament and the Council for animal experiments [70].

### 3.2. Isolation of Spleen Mononuclear Cells (SMNCs)

The organisms were euthanized by cooling, followed by freezing (a method approved by the Institutional Animal Care at the University of Oregon) [71], and the spleen was immediately dissected aseptically. The spleens were then passed through a 100 mm nylon mesh and disaggregated with gentle movements using a syringe plunger. Spleen mononuclear cells (SMNCs) were separated by density gradient (5 mL cell suspension in 2.5 mL Histopaque-1077) at 1000× *g* at 20 °C for 20 min. The SMNCs were recovered at 1900× *g* for 15 min, and the cellular pellet was reconstituted in 1 mL RPMI-1640 medium.

SMNCs were characterized through flow cytometry based on size (forward scatter, FSC) and cellular complexity (side scatter, SSC) with a BD Accuri C6 flow cytometer (BD Becton Dickinson, San Jose, CA, USA). After establishing the study cell population, a cell count was performed. Prior to cell culture, cell viability was assessed using an Annexin V-FITC Kit (BD Pharmingen™, Franklin Lakes, NJ, USA) [31].

### 3.3. In Vitro Exposure to Diazoxon and Cholinergic Agonists and Antagonists

SMNCs (4 × 10^5^) were cultured in RPMI-1640 medium supplemented with 10% bovine serum albumin (BSA) and 1% streptomycin/penicillin in 24-well plates at 28 °C, with 95% air and 5% CO_2_ for 24 h. Afterwards, cells were exposed to cholinergic agonists and antagonists. Nicotine (1 μM/5 min) served as an nAChR agonist, and bungarotoxin (1 μM/10 min) was used as an nAChR antagonist. Carbachol (1 μM/10 min) and atropine (1 μM/30 min) were employed as the mAChR agonist and antagonist, respectively. The concentrations of the agonists and antagonists were determined based on dose–response curves using Ca^2+^ flux measured by flow cytometry [31].

For the pesticide assays, cells were exposed to 0.01 μM, 1 μM, and 10 μM of DXN (reagent grade, 97.3% purity, Chem Service, West Chester, PA, USA) for 24 h at 28 °C, with 95% air and 5% CO_2_. DXN concentrations for in vitro assays were selected based on the percentage of inhibition of the AChE enzyme (100% activity, ±50% activity, and <10% activity, respectively) [31,32].

### 3.4. Master Transcription Factors Expression

Total RNA was isolated from cultured SMNCs following the method described by Rio et al. [72]. The integrity and purity of the RNA were evaluated using gel electrophoresis and measured by UV spectrophotometry at 260/280 nm. cDNA was synthesized with a TaqMan™ Reverse Transcription Kit (Applied Biosystems, Waltham, MA, USA) according to the manufacturer’s instructions. Gene expression analysis was performed by real-time PCR using a 7500 Fast Real-Time PCR System (Applied Biosystems, Waltham, MA, USA) with Fast SYBR Green Master Mix (Life Technologies, Carlsbad, CA, USA) [32]. For expression analysis, the primers of the target genes (T-bet, GATA3, Foxp3, and RORγ) and the housekeeping gene (elongation factor-1 alpha, EF1-α) are shown in Table 1.

The PCR protocol included an initial denaturation at 94 °C for 4 min, followed by 35 amplification cycles comprising 94 °C for 30 s, 64 °C for 30 s, and 72 °C for 30 s. A final extension step at 72 °C lasted 10 min. Each sample was run in triplicate to ensure accuracy. Melting curve analysis was performed at the end of each PCR to verify the specificity of the amplified product. Relative gene expression levels were calculated using the 2^−ΔΔCT^ method [75].

### 3.5. Statistical Analysis

Statistical analysis was conducted using one-way ANOVA followed by Tukey’s multiple comparisons test to assess differences among group means. Before applying parametric tests, data were evaluated for normality with the Shapiro–Wilk test and for homogeneity of variances using Levene’s test. All experiments included n = 7 independent biological replicates per group. A *p*-value of 0.05 or lower was considered statistically significant. All analyses were performed with GraphPad Prism version 9.2.0 (GraphPad Software, San Diego, CA, USA).

## 4. Conclusions

Therefore, the results presented here support the idea that alterations in the master transcription factor expression of Nile tilapia leukocytes occur through cholinergic stimulation (both muscarinic and nicotinic), probably directing the immune response toward a pro-inflammatory profile primarily regulated by the RORγ and T-bet transcription factors. However, variation in responses to different stimuli may be influenced by numerous factors discussed in this paper, such as the cholinergic receptor types expressed per cell, activation of their signaling pathways, factors affecting receptor expression, and the ligand-binding affinities, including the dose and duration of exposure to the agonist, antagonist, or pesticide, among others. Further research to clarify these molecular mechanisms of immune regulation via the non-neuronal cholinergic system is important.

## Figures and Tables

**Table 1:** Oligonucleotide sequences used for expression analysis through RT-qPCR.

Gene	Forward	Reverse	Reference
T-bet	5′CCTCCTCATCTTCTACATCAC 3	5′CCTCTTCTTGTTTTCACCCAC3′	Yao et al., 2019 [73]
GATA-3	5′CTGGAGGGGAGCAAAGGAAT3′	5′CGTGAAGAGGTGTGGACTGG3′	Yao et al., 2019 [73]
Foxp3	5′GCAGCCTCAGGTTACCACTG3′	5′AAGGAGGCCTGATGTTGTTG3′	Wei et al., 2013 [21]
RORγ	5′CCATGGAGGTGGTTCTGGTC 3′	5′CAGGGCACTGAAGAGAGCAA 3′	Primer-BLAST (NCBI)
EF1-α	5′CAAGGAAATCCGTCGTGGATAC3′	5′ACGGCGAAACGACCGAGGGG3′	Yang et al., 2013 [74]

## Data Availability

The raw data supporting the conclusions of this article will be made available by the authors on request.
